# Alfalfa and barley association promote the ability of plant growth-promoting microbes to mitigate drought and salt stresses

**DOI:** 10.3389/fpls.2025.1646620

**Published:** 2025-10-13

**Authors:** Aiman Slimani, Martin Jemo, Khalid Oufdou, Abdelilah Meddich

**Affiliations:** ^1^ AgroBiosciences Program, College for Sustainable Agriculture and Environmental Sciences, University Mohammed VI Polytechnic (UM6P), Ben Guerir, Morocco; ^2^ Laboratory of Water Sciences, Microbial Biotechnologies, and Natural Resources Sustainability (AQUABIOTECH), Unit of Microbial Biotechnologies, Agrosciences, and Environment (BIOMAGE)-CNRST Labeled Research Unit N°4, Faculty of Sciences Semlalia, University Cadi Ayyad, Marrakech, Morocco; ^3^ Center of Agrobiotechnology and Bioengineering, Research Unit Labeled CNRST (Centre AgroBiotech-URL-CNRST-05), “Physiology of Abiotic Stresses” Team, Cadi Ayyad University, Marrakech, Morocco; ^4^ African Sustainable Agriculture Research Institute (ASARI), College for Sustainable Agriculture and Environmental Sciences, University Mohammed VI Polytechnic (UM6P), Laayoune, Morocco

**Keywords:** antioxidant activity, cascading effect, growth, physiology, osmolytes, stress markers

## Abstract

The advantages of crop association, or intercropping, include reducing disease cycles, suppressing weeds, and enhancing nutrient transfer between crops, both above and below-ground, are well-studied. However, the potential of associated crops to alter their physiology through interactions with rhizosphere microbes, which could mitigate drought and salt stresses, is underexplored. We investigated the impact of combining alfalfa (*Medicago sativa*) and barley (*Hordeum vulgare*) with or without plant growth-promoting microbes (PGPM) and compost on growth, physiology, stress markers, osmolytes, and antioxidant enzymes under drought and salt stress. Alfalfa and barley seedlings were grown either as sole crop or in combination, and treated with one of the biological options, including rhizobacteria (R) or mycorrhizal (M) consortia, along with compost (C) amendments, or with one of the combinations RM, RC, MC, and MRC. The seedlings were subjected to combined salt and drought stress, as well as control conditions without stress. Stress-induced markers, including malondialdehyde (MDA) and hydrogen peroxide (H_2_O_2_) levels, osmolyte accumulation (total sugars and proteins), enzymatic antioxidant activities such as superoxide dismutase (SOD) and catalase (CAT), as well as growth, total chlorophyll, and stomatal conductance in leaf tissues, were measured at harvest. The effect of associating alfalfa and barley in the same pot resulted in highly significant effects on the shoot dry weight, H_2_O_2_, protein, MDA, and sugar contents compared to a sole cultivation of alfalfa and barley. Stressed plants showed higher levels of MDA, sugar content, and SOD activity, regardless of the crop combination. Among the biological treatments, the MRC disclosed the highest shoot dry weight, sugar content, and SOD activity for the alfalfa under crop association. Correspondence analysis with forward selection of the functional variables’ importance revealed that total chlorophyll (54.4%) and protein content (15.0%) accounted for a significant portion of the dataset’s variability. We discuss belowground biotic benefit effects of intercropping in managing abiotic stress, boosting resilience in arid systems, and promoting sustainable agricultural practices.

## 1 Introduction

Crop association, also referred to as intercropping in the field context, is a crop diversification strategy that involves simultaneously cultivating two or more crop species in the same field, thereby enhancing aboveground productivity and crop diversity, and representing a compelling opportunity for the sustainable intensification of agriculture (Dai et al., 2019). The above-ground benefits of intercropping include reducing disease incidence caused by pathogens, increasing light interception, and suppressing weeds (Zhang et al., 2019; Li et al., 2024). At the belowground level, nutrient transfer between associated crops is one of the primary benefits of intercropping. It further drives positive microbiota enrichments between the crop species in the rhizosphere and contributes to carbon sequestration governed by associated crops and microorganisms (Domeignoz-Horta et al., 2024). Another benefit of crop association is that it maximizes root system expansions and stimulates rhizosphere processes, such as increased root exudates and signaling molecules through specific interactions (Li et al., 2024). In a traditional cereal-legume intercropping system at the field level, legume species are usually grown on top of the mount and the cereal at the bottom to benefit from the N rhizodeposition through the N_2_ fixation process (Ma et al., 2024). A recent study highlights the cascading biotic interactions between intercropped species, resident microbiomes, and crop species, promoting resilient farming practices (Li et al., 2024). However, due to the increased frequency of dry spells exacerbated by climate change’s acceleration, plants are more exposed to drought and salt stresses (Yavas et al., 2024). Consequently, the abovementioned intercropping benefits in alleviating these abiotic stresses are under-investigated, particularly in the arid regions of the Mediterranean.

Globally, abiotic stresses, particularly drought, heat, and salinity, account for 50% to 90% of crop yield reductions (Toulotte et al., 2022). Drought stress alone often causes a decrease of up to 50% in cereal grain yield, depending on the phase of the drought, which may be terminal or severe (Rezaei et al., 2023), while legume yield decreases by 24 to 87% during the reproductive period under severe and terminal drought conditions are reported (Khatun et al., 2021). Furthermore, soil salinity also leads to decreases in crop yields, ranging from 13.3% to 58.3% (Haj-Amor et al., 2022). Drought and salt stress alter several plant functions, including photosynthesis, energy metabolism, water balance, and nutrient uptake, ultimately affecting crop growth and yield (Ors et al., 2021). Plants exposed to drought and salt stresses exhibit an increased accumulation of reactive oxygen species (ROS), including malondialdehyde (MDA) and hydrogen peroxide (H_2_O_2_), which causes degradation of the tissue phospholipid double layer of membranes and plant lethality if prolonged (Wang et al., 2025). In response, plants reduce ROS accumulation by activating primary and secondary metabolites, leading to increased production of sugars and proteins that scavenge increased ROS levels and provide systemic responses against abiotic stresses (Thiruvengadam et al., 2024).

There is great interest in using ecologically acceptable and low-cost management interventions to tackle abiotic stresses that cause oxidative stress. The use of beneficial microbes and organic amendments to alleviate environmental stresses is gaining increased attention as a practical tool for enhancing crop growth and productivity. Organic amendments, such as compost, increase plant resilience by improving soil water retention, increasing soil organic matter, and enhancing nutrient availability, thereby improving crop growth and productivity (Ho et al., 2022). Additionally, plant growth-promoting microbes (PGPM), including arbuscular mycorrhizal fungi (AMF) and plant growth-promoting rhizobacteria (PGPR), play a crucial role in enhancing plant growth and resilience to environmental stress (Brokate et al., 2024; Panda and Das, 2024). AMF can assist plant growth by enhancing water absorption and mineral uptake, particularly from challenging-to-reach soil niches via their hyphal network (Slimani et al., 2024). PGPR plays a vital role in promoting plant growth by enhancing nutrient absorption through atmospheric di-nitrogen fixation, solubilizing essential nutrients such as phosphorus (P) and potassium (K), and chelating iron (Fe) via siderophores (Khoso et al., 2024). PGPR can also help plants manage abiotic stress by synthesizing aminocyclopropane-1-carboxylic acid (ACC) deaminase, which reduces ethylene levels and produces phytohormones such as indole acetic acid (IAA) and gibberellins. This process enhances the plant’s immune response and strengthens signal transduction, leading to optimal development (Khoso et al., 2024).

The world’s population is expected to increase and reach approximately 9.7 billion by 2050, requiring a 70% increase in food production to meet demand (Ammar et al., 2024). This challenge is further exacerbated by climate change. By 2050, more than 50% of arable land will be subjected to periodic droughts with considerable impacts, affecting agricultural systems (Lamrabet et al., 2024). Approximately 20% of all cultivated land worldwide and 33% of irrigated agricultural land are affected by salinization (Shrivastava and Kumar, 2015). Furthermore, it is estimated that salinized land will expand at an annual rate of 10%, with projections indicating that it could englobe 50% of arable land by 2050 (Shrivastava and Kumar, 2015).

Implementing a resiliency system that promotes resource complementarity, such as a well-known legume-cereal association benefit system coupled with affordable biological methods, can be an effective strategy to enhance crop productivity in the face of climate change-related abiotic stresses and address increasing food needs. This study aimed to assess the effectiveness of combining an intercropping system with biological treatments in mitigating the harmful impacts of simultaneous drought and salt stress. Specifically, we aimed to:

- Assess how the association of alfalfa and barley alters the physiology of each crop compared to the sole crops of alfalfa or barley cropping practices, and in the presence or absence of inoculated microbes to mitigate drought and salinity stresses
- Investigate biological combinations that enhance crop resilience to drought and salt stress while improving osmolyte accumulation and activating the enzymatic antioxidant system.
- Highlight the rhizosphere biotic interaction mechanisms shaped by the cascading effects of crop species and beneficial microbes, which alleviate abiotic stress and promote sustainable farming practices.

## 2 Materials and methods

### 2.1 Biological materials

Seeds of *Hordeum vulgare* (var. Amalou) obtained from the Société Nationale de Commercialisation des Semences (SONACOS), Meknès, and a commercial landrace variety of *Medicago sativa* (var. Demnate) were selected for this experiment. The seeds were surface-disinfected and pre-germinated.

The microbial inocula used in this study consisted of an AMF consortium isolated from the rhizospheric soil of *Opuntia ficus-indica* and containing seven genera and 22 species (Slimani et al., 2023). The PGPR consortium was composed of different rhizobacterial strains (*Pantoea agglomerans*, *Pseudomonas zanjanensis*, *Streptomyces swartbergensis*, and *Streptomyces cahuitamycinicus*) isolated from the rhizospheres of *H. vulgare* and *M. sativa* in the Marrakech-Safi region, Morocco. The two rhizobia species/strains were isolated from *M. sativa* nodules grown in soils from semi-arid regions and identified as *Ensifer meliloti* (Slimani et al., 2023). Consortia and the inoculation method were prepared using the Slimani et al. (2023) protocol. The mature organic amendment, produced from animal and plant wastes, was applied profusely before transplanting seedlings at a concentration of 2% (w/w: compost/soil) (**Table 1**).

**Table 1:** Physico-chemical properties of the used soil and compost.

Parameters	Soil	Compost
Sand (%)	51.00	–
Clay (%)	19.00	–
Loam (%)	30.00	–
pH	8.60	8.99
Total organic carbon (%)	0.59	19.45
Total N concentration (g kg^-1^)	1.1	20.7
Available P concentration (mg kg^-1^)	11	1190
Total K concentration (g kg^-1^)	0.75	0.92

The soil used to grow and establish the pot experiment was sampled at 0–20 cm layer from an agricultural field in the peri-urban area of Marrakech (31°39’07.3”N, 8°04’04.3”W). Several cores of soil samples were collected and bulked to form a composite sample, which was then passed through a 4 mm sieve and autoclaved. The analyzed physicochemical characteristics of the soil are reported in **Table 1**.

### 2.2 Experimental design

A factorial design was implemented with three main factors and eight replicates under greehouse conditions: factor 1: crop association (Ca) at three levels: sole barley (1 barley seed per pot), sole alfalfa (3 alfalfa seeds per pot) and alfalfa-barley association (1 barley seed + 3 alfalfa seeds per pot), factor 2: environmental stress (Es): no-stress conditions (plants were not subjected to any salt stress and were watered at a level of 75% of field capacity (FC)) and stressed conditions (plants subjected to simulated drought and salt stress, they were subjected to a salt treatment with 120 mM and a watering level of 35% of FC), and factor 3: biological treatments (Bt) at eight levels: (1) Control (Con): plants without any biological treatment, (2) C: plants treated with compost; (3) M: plants treated with AMF consortium; (4) R: plants treated with bacterial consortium; (5): MC: plants treated with AMF consortium and compost; (6): RC: plants treated with bacterial consortium and compost; (7): MR: plants treated with AMF consortium and bacterial consortium; and (8) MRC: plants treated with AMF consortium, bacterial consortium, and compost; were used to establish the experiment. The pots were placed in a controlled culture chamber featuring a 16-hour light and 8-hour dark cycle, with a light intensity of 580 μmol m^-2^ s^-1^, maintaining a relative humidity of 60% and an average temperature of 25°C.

### 2.3 Growth and mycorrhization measurements

At harvest, 120 days after sowing, the plants were carefully uprooted to avoid damage or loss of roots and gently washed with distilled water to remove any adhering substances. Total biomass dry weight was obtained after dehydrating at 80°C for three days. Fresh roots were delicately cut into 1 cm fragments, cleaned with 10% KOH at 90°C for 30 minutes, and then exposed to 2% HCl for 10 minutes. The roots were stained with 0.05% trypan blue at 90°C for 30 minutes (Phillips and Hayman, 1970). Microscopic analysis was conducted using a ZEISS Axioskop 40 microscope at magnifications ranging from 40 to 100 (Carl Zeiss; Oberkochen, Germany). The frequency (F%) and intensity (I%) of root mycorrhizal infection were determined as referred to Trouvelot et al. (1986).

### 2.4 Stomatal conductance (gs) and total chlorophyll concentration

The gs was measured on fully developed leaves from the apex using a Leaf Porometer (LP1989) (Decagon Device, Inc., Washington, DC, US). The chlorophyll extract was obtained from leaf tissues following the Arnon (1949) procedure. The absorbance of the supernatant was measured at two specific wavelengths, 645 *η*m and 663 *η*m, using a double-beam spectrophotometer (VWR, UV-6300PC, China). The Chl T concentration was calculated using the following equation:


$$\text{Chl T}=\frac{(20.2\times \text{A}645)+(8.02\times \text{A}663)\times \text{V}}{1000\text{ FW}}$$

Where: V: final volume made, and FW: fresh weight.

### 2.5 Determination of MDA and H_2_O_2_

The MDA content was measured using the method described by Horchani et al. (2023). Fresh leaves weighing 0.25 g were homogenized in 10 mL of 0.1% trichloroacetic acid (TCA) and then centrifuged at 18,000 rpm for 20 minutes. The resulting supernatant (1 mL) was mixed with 2 mL of 20% TCA containing 0.5% thiobarbituric acid (TBA) and heated to 100°C for 30 minutes. The mixture was subsequently cooled in an ice bath. Absorbance readings were taken at 450 nm, 532 nm, and 600 nm. The H_2_O_2_ content was estimated using the method of Velikova et al. (2000). Briefly, 0.25 g of fresh leaves were homogenized in a cold mortar with 5 mL of 10% (w/v) TCA, followed by centrifugation at 15,000 rpm for 15 minutes at 4°C. Next, 0.5 mL of the recovered supernatant was mixed with 0.5 mL of 1 mM phosphate buffer (pH 7.0) and 1 mL of 1M potassium iodide. After one hour of incubation, the absorbance at 390 nm was measured, and the H_2_O_2_ determined using a standard curve.

### 2.6 Soluble sugar, and total protein contents

The soluble sugar content were determined according to the method described by DuBois et al. (1956). A 0.1 g sample of frozen leaf was homogenized with 8 mL of 80% ethanol, followed by centrifugation at 5,000 rpm for 10 minutes. The supernatant obtained (0.2 mL) was then combined with 0.2 mL of 5% phenol and 1 mL of concentrated sulfuric acid. After a 10-minute cooling period, the absorbance was measured at 485 nm. In a cold mortar, 0.1 g of frozen leaf powder samples were homogenized with 4 mL of 0.1M phosphate buffer (pH 6.0) containing 5% polyvinylpolypyrrolidone (PVPP) and 0.1mM ethylene diamine tetraacetic acid (EDTA). After centrifugation at 18,000 rpm for 10 minutes at 4°C, the supernatant was used to determine the total protein content and the activities of various antioxidant enzymes. Total protein content was assessed using the method described by Bradford (1976). Briefly, 5 mL of Bradford’s reagent was added to 0.1 mL of the extract. The reaction mixture was homogenized and incubated at 30°C for 30 minutes. The absorbance was then measured using a spectrophotometer at 595 nm.

### 2.7 Superoxide dismutase (SOD) and catalase (CAT) activities

The SOD activity was assessed by monitoring the nitro-blue tetrazolium (NBT) reduction spectrophotometrically at 560 nm after 30 minutes under blue light, with one unit defined as the amount of enzyme required to cause a 50% inhibition of NBT reduction (Rekik et al., 2017). The reaction mixture included 300 µL of 0.75 mM NBT, 2,550 µL of 0.1 mM phosphate buffer (pH 6.0), 60 µL of 0.1 mM riboflavin, 75 µL of 55 mM methionine, and 50 µL of enzyme extract. Control tests were conducted in the absence of substrate to evaluate the auto-oxidation of the substrates. The CAT activity was assessed using the method of Duarte et al. (2015). The reaction mixture consisted of 890 μL of 0.1 M phosphate buffer (pH 7.0) and 100 μL of enzyme extract, with the reaction initiated by the addition of 10 μL of 15% H_2_O_2_. Enzyme activity was calculated by measuring the decrease in absorbance at 240 nm over time.

### 2.8 Statistical data analysis

The effects of crop association (Ca), environmental stress (Es), biological treatments (Bt), and their interactions were examined through three-way, two-way, or one-way analyses of variance (ANOVA) using the General Linear Procedure (GLM) in JMP statistical software, version 14 (2022). The Least-Means/PDIFF option was used to separate means among Ca, Es, and Bt. This was carried out exclusively when the Student’s *t*-test from ANOVA was significant at *p* < 0.05. Levels of significance in Tables are given by ns (not significant, *p* ≥ 0.05); *p* < 0.05; **p < 0.01; ***p < 0.001. Correspondence analysis (CCA) was conducted to extract patterns of variation from the canonical axes and functional traits. The tests of significance for both canonical axes were carried out, and the *p*-values reported represent the significance determined by the Monte Carlo permutation with 499 permutations. A forward selection of the functional variables was implemented, and variables that significantly contributed to the axes are reposted on the biplots. The hierarchical heat map of the entire dataset, crop association, and sole cropping was computed using “*phylo. makers” functions in the ‘*phyr’ packages (RStudio Team, 2024). We considered the measured variable for the two years and each year separately. The table showing the correlation between pairs of functional variables was calculated and displayed.

## 3 Results

### 3.1 Photosynthesis activities and mycorrhizal colonization

**Supplementary Table S1** presents the analysis of variance testing the effects of crop association (Ca), environmental stress (Es), and biological treatments (Bt) on gs, Chl T, and mycorrhizal colonization frequency and intensity. The effects of Ca, Es, and Bt were highly significant at *p* < 0.001, regarding the gs, ChlT, AMF colonization frequency, and intensity. The non-stressed plants grown in association with barley exhibited higher Chl T and gs contents than the stressed and non-stressed sole cultivated alfalfa under the different biological combinations (**Supplementary Table S2**). Regarding barley, we found that plants grown alongside alfalfa exhibited higher Chl T and gs levels than barley grown alone, under both stressed and non-stressed conditions across various Bt treatments (**Supplementary Table S2**). The frequency of mycorrhizal colonization ranged from 0.0 ± 0.00 to 98.3 ± 1.27. Lower colonization frequencies were observed in controls, C, R, RC, and R treatments across various alfalfa and barley combinations, as well as sole crops of both non-stressed and stressed plants (**Supplementary Table S2**). The highest frequency value was observed in the MRC treatment of barley cultivated simultaneously with alfalfa under the non-stressed treatment (**Supplementary Table S3**). The mycorrhizal colonization intensity was the highest in the MRC treatment of barley cultivated simultaneously with alfalfa under the non-stressed treatment (**Supplementary Table S2**). The interactions between the Ca × Es and Ca × Bt were highly significant for the Chl T, mycorrhizal colonization frequency, and intensity, respectively. We also observed a significant interaction between Es and Bt for Gs and Chl T, respectively.

### 3.2 Shoot growth, H_2_O_2_ and protein contents, and CAT activity

The results of the three-way ANOVA test for Ca, Es, Bt, and their interactions on shoot dry weight, H_2_O_2_, protein content, and CAT activity are shown in **Table 2**. The effects of Es and Bt were highly significant (*p* < 0.001) regarding the shoot dry weight, H_2_O_2_, protein content, and CAT activity (**Table 2**). The Ca effect was significant on the shoot dry weight, H_2_O_2_, and protein content, but not on the CAT activity. The tested interactions (Ca × Es, Ca × Bt, Es × Bt, and Ca × Es × Bt) were highly significant for H_2_O_2_. The interaction between Ca and Es was significant for shoot dry weight. We noted a significant interaction between Es and Bt on the shoot dry weight and protein content (**Table 2**). The shoot dry weight of alfalfa and barley in association with non-stressed conditions was higher than that of plants in sole pots under the different Bt conditions (**Table 3**). The MRC treatment of the stressed alfalfa and barley plants showed the highest shoot dry weight compared to stressed sole alfalfa and barley pots (**Table 3**). Stressed sole-planted barley plants exhibited higher H_2_O_2_ content under the control treatment than other biological treatments and associated plants (**Table 3**). The MRC and MR treatments demonstrated the lowest H_2_O_2_ contents compared to other treatments (**Table 3**). The protein content was highest in stressed alfalfa and barley grown together compared to non-stressed plants, as well as in sole alfalfa and barley, respectively. Barley exhibited a higher activity of CAT than alfalfa. The stressed alfalfa plants, in association, demonstrated higher activity of CAT than sole-planted plants. The MRC treatment showed the highest CAT activity in both alfalfa and barley plants.

**Table 2:** Three-way analysis of variance (ANOVA) testing the effects of crop association (Ca), environmental stress (Es), biological treatments (Bt), and their interactions on the shoot dry weight, hydrogen peroxide, protein content, and catalase activity.

Source of variation	DF	Shoot dry weight (g plant^-1^)			Hydrogen peroxide (nmol g^-1^ FW)		
		*F value*	*p value*	*Sig.*	*F value*	*p value*	*Sig.*
Crop association (Ca)	1	151.3	<0.001	(***)	1914.0	<0.001	(***)
Environmental Stress (Es)	1	762.1	<0.001	(***)	47003.3	<0.001	(***)
Biological treatment (Bt)	7	130.1	<0.001	(***)	9130.2	<0.001	(***)
Ca × Es	1	42.2	<0.001	(***)	75.7	<0.001	(***)
Ca × Bt	7	1.2	0.3161	ns	5.3	<0.001	(***)
Es × Bt	7	3.1	0.004	(**)	2206.6	<0.001	(***)
Ca × Es × Bt	7	0.2	0.9845	ns	4.2	<0.001	(***)

Source of variation	DF	Protein content (mg g^-1^ FW)			Catalase activity (EU mg^-1^ protein min^-1^)		
		*F value*	*p value*	*Sig.*	*F value*	*p value*	*Sig.*
Crop association (Ca)	1	411.6	<0.001	(***)	2.41	0.12	ns
Environmental Stress (Es)	1	10396.8	<0.001	(***)	74.94	<0.001	(***)
Biological treatment (Bt)	7	1101.8	<0.001	(***)	4.00	<0.001	(***)
Ca × Es	1	0.1	0.71	ns	0.02	0.89	ns
Ca × Bt	7	0.5	0.84	ns	0.02	1.00	ns
Es × Bt	7	20.7	<0.001	(***)	0.05	1.00	ns
Ca × Es × Bt	7	1.3	0.23	ns	0.00	1.00	ns

DF: degree of freedom. ns, not significant; *Significant at *p* < 0.05; **Significant at *p* < 0.01; ***Significant at *p* < 0.001.

**Table 3:** Effect of crop association, environmental stress, and biological treatments on the shoot dry weight, hydrogen peroxide, protein content, and catalase activity of alfalfa and barley plants.

	Alfalfa in association		Sole alfalfa		Barley in association		Sole barley	
	Non stressed	Stressed	Non stressed	Stressed	Non stressed	Stressed	Non stressed	Stressed
Shoot dry weight (g plant^-1^)								
Con	0.53 ± 0.009 gC	0.21 ± 0.010 hG	0.34 ± 0.013 hE	0.13 ±0.007 hH	1.21 ± 0.010 hA	0.37 ±0.008 hD	0.82 ±0.012 gB	0.29 ±0.009 hF
C	0.75 ± 0.011 fC	0.28 ± 0.008 gG	0.43 ± 0.008 gE	0.19 ± 0.008 gH	1.32 ± 0.010 gA	0.48 ± 0.009 gD	0.92 ± 0.010 fB	0.39 ± 0.008 gF
M	0.86 ± 0.010 eC	0.39 ± 0.012 fF	0.49 ± 0.009 fE	0.30 ± 0.008 fG	1.45 ± 0.008 fA	0.59 ±0.008 fD	1.11 ±0.018 eB	0.49 ± 0.010 fE
R	1.14 ± 0.016 dC	0.49 ± 0.011 eF	0.74 ± 0.011 eD	0.34 ±0.008 eG	1.93 ± 0.018 eA	0.75 ±0.011 eD	1.41 ±0.020 dB	0.65 ±0.011 eE
MC	1.16 ± 0.012 dC	0.59 ± 0.011 dF	0.86 ± 0.011 dD	0.41 ±0.011 dG	2.06 ± 0.011 dA	0.87 ±0.014 dD	1.45 ±0.012 cdB	0.74 ± 0.010 dE
RC	1.37 ± 0.018 cC	0.66 ± 0.008 cG	0.99 ± 0.016 dE	0.55 ±0.013 cH	2.16 ± 0.009 cA	1.06 ±0.011 cD	1.48 ±0.015 cB	0.89 ±0.011 cF
MR	1.61 ± 0.010 bC	0.74 ± 0.011 bG	1.17 ± 0.016 bE	0.61 ±0.004 bH	2.23 ± 0.019 bA	1.22 ±0.011 bD	1.67 ±0.016 bB	1.07 ± 0.009 bF
MRC	1.91 ± 0.015 aB	1.16 ± 0.012 aE	1.42 ± 0.015 aD	0.87 ±0.015 aF	2.51 ± 0.017 aA	1.66 ±0.014 aC	1.91 ±0.018 aB	1.42 ± 0.015 aD
Hydrogen peroxide (nmol g^-1^ FW)								
Con	16.3 ± 0.12 aG	43.2 ± 0.12 aD	18.5 ± 0.11 aE	47.3 ± 0.12 aA	16.0 ± 0.12 aG	42.2 ± 0.09 aD	17.6 ± 0.14 aF	45.5 ± 0.11 aB
C	14.7 ± 0.09 bF	30.3 ± 0.09 bC	16.7 ± 0.09 b	34.5 ± 0.11 bA	13.4 ± 0.10 bG	30.2 ± 0.09 bC	15.6 ± 0.10 bF	33.3 ± 0.08 bB
M	13.1 ± 0.10 cG	24.6 ± 0.09 cC	15.4 ± 0.11 cE	27.8 ± 0.09 cA	11.9 ± 0.11 cH	23.4 ± 0.09 cD	13.6 ± 0.13 cG	26.4 ± 0.09 cA
R	9.4 ± 0.10 dH	20.4 ± 0.09 dC	11.4 ± 0.10 dE	23.5 ± 0.09 dA	9.1 ± 0.12 dG	19.2 ± 0.10 dD	10.7 ± 0.12 dF	21.6 ± 0.09 dB
MC	8.6 ± 0.11 eG	16.2 ± 0.08 eC	10.3 ± 0.09 eE	19.3 ± 0.09 eA	7.9 ± 0.09 eH	15.7 ± 0.09 eD	9.6 ± 0.12 eF	18.4 ± 0.08 eB
RC	7.6 ± 0.08 fG	14.3 ± 0.10 fC	9.4 ± 0.10 fE	17.7 ± 0.08 fA	6.7 ± 0.10 fH	13.7 ± 0.09 fD	8.9 ± 0.10 fF	16.3 ± 0.10 fB
MR	6.5 ± 0.11 gF	13.5 ± 0.09 gC	8.3 ± 0.10 gE	16.3 ± 0.09 gA	6.2 ± 0.12 gG	12.3 ± 0.10 gD	8.2 ± 0.12 gE	14.4 ± 0.10 gB
MRC	5.3 ± 0.09 hG	11.3 ± 0.08 hC	7.3 ± 0.09 hE	13.2 ± 0.09 hA	5.7 ± 0.09 hF	10.4 ± 0.08 hD	7.5 ± 0.10 hE	11.7 ± 0.10 hB
Protein content (mg g^-1^ FW)								
Con	18.8 ± 0.35 hF	54.5 ± 0.35 hB	12.4 ± 0.35 hH	44.1 ± 0.29 hD	21.0 ± 0.34 hE	56.3 ± 0.34 hA	14.0 ± 0.41 hG	46.2 ± 0.35 hC
C	24.4 ± 0.32 gF	85.5 ± 0.34 gA	16.5 ± 0.34 gH	76.6 ± 0.31 gB	31.6 ± 0.35 gE	70.5 ± 0.33 gC	21.7 ± 0.36 gG	64.2 ± 0.36 gD
M	35.9 ± 0.32 fF	96.4 ± 0.35 fA	22.7 ±0.35 fH	87.4 ± 0.35 fB	37.9 ± 0.36 fE	80.8 ± 0.35 fC	26.8 ± 0.34 fG	71.8 ± 0.33 fD
R	53.9 ± 0.36 eE	107.7 ± 0.34 eA	43.5 ± 0.34 eG	98.2 ± 0.37 eB	54.5 ± 0.35 eE	94.1 ± 0.35 eC	44.7 ± 0.35 eF	85.4 ± 0.36 eD
MC	58.0 ± 0.36 dE	108.8 ± 0.37 dA	43.5 ± 0.37 dF	105.2 ± 0.35 dB	58.5 ± 0.34 dE	102.5 ± 0.38 dC	48.4 ± 0.33 dF	92.1 ± 0.34 dD
RC	62.0 ± 0.35 cE	120.8 ± 0.35 cA	56.0 ± 0.36 cF	108.9 ± 0.36 cB	62.9 ± 0.32 cE	107.8 ± 0.34 cC	56.0 ± 0.31 cF	96.7 ± 0.36 cD
MR	69.5 ± 0.34 bF	123.5 ± 0.39 bA	59.1 ± 0.28 bH	113.1 ± 0.38 bB	70.6 ± 0.33 bE	107.8 ± 0.33 bC	60.2 ± 0.35 bG	98.3 ± 0.31 bD
MRC	76.6 ± 0.34 aF	134.0 ± 0.38 aA	66.5 ±0.35 aH	122.6 ± 0.33 aB	83.4 ± 0.33 aE	113.8 ± 0.41 aC	71.1 ± 0.34 aG	106.6 ± 0.35 aD
Catalase activity (EU mg^-1^ protein min^-1^)								
Con	5.6 ± 0.10 hG	12.4 ± 0.09 hE	4.5 ± 0.10 gH	10.3 ± 0.09 hF	33.9 ± 0.14 hC	73.6 ± 0.14 hA	27.8 ± 0.17 hD	68.5 ± 0.15 hB
C	6.7 ± 0.08 gG	13.5 ± 0.08 gE	5.6 ± 0.07 fH	12.0 ± 0.10 gF	36.6 ± 0.13 gC	76.8 ± 0.11 gA	30.4 ± 0.14 gD	71.8 ± 0.11. gB
M	8.7 ± 0.09 fG	14.9 ± 0.09 fE	6.8 ± 0.11 eH	12.7 ± 0.10 fF	39.8 ± 0.14 fC	79.7 ± 0.12 fA	34.3 ± 0.13 fD	74.5 ± 0.11 fB
R	10.5 ± 0.10 eG	16.2 ± 0.10 eE	8.7 ± 0.09 dH	14.6 ± 0.10 eF	49.2 ± 0.13 eC	82.7 ± 0.10 eA	44.7 ± 0.19 eD	78.7 ± 0.10 eB
MC	11.6 ± 0.08 dG	17.9 ± 0.10 dE	9.7 ± 0.10 cH	16.3 ± 0.10 d F	53.5 ± 0.13 dC	86.9 ± 0.11 dA	47.6 ± 0.11 dD	83.4 ± 0.10 dB
RC	12.0 ± 0.10 cG	18.5 ± 0.10 cE	10.3 ± 0.08 bH	16.9 ± 0.10 cF	57.7 ± 0.11 cC	91.3 ± 0.11 cA	51.0 ± 0.10 cD	86.3 ± 0.11 cB
MR	12.8 ± 0.09 bG	19.6 ± 0.09 bE	10.5 ± 0.09 bH	17.5 ± 0.10 bF	60.5 ± 0.14 bC	92.0 ± 0.10 bA	52.2 ± 0.13 bD	87.5 ± 0.11 bB
MRC	14.6 ± 0.12 aG	26.8 ± 0.11 aE	12.9 ± 0.10 aH	23.3 ± 0.11 aF	71.3 ± 0.11 aC	101.3 ± 0.09 aA	60.6 ± 0.12 aD	93.2 ± 0.12 aB

Means (n=8) ± s.e within the same plant followed by a different letter are statistically different (*p* < 0.001) following Tukey’s. Con, control; M, arbuscular mycorrhizal fungi consortium; R, bacterial consortium; C, compost; MC, arbuscular mycorrhizal fungi consortium and compost; RC, bacterial consortium and compost; MR, arbuscular mycorrhizal fungi consortium and bacterial consortium; MRC, arbuscular mycorrhizal fungi consortium; bacterial consortium, and compost. Different lower-case letters denote significant differences among the biological treatments under non-stressed, stressed, and association types (Alfalfa, barley, or sole alfalfa and sole barley) at Fisher’s test *p* < 0.05. Different capital letters denote significant differences within each biological treatment among the non-stressed, stressed, and association types (Alfalfa, barley, or sole alfalfa and sole barley) at Fisher’s test *p* < 0.05.

### 3.3 MDA, sugar content, and SOD activity

**Figure 1:**
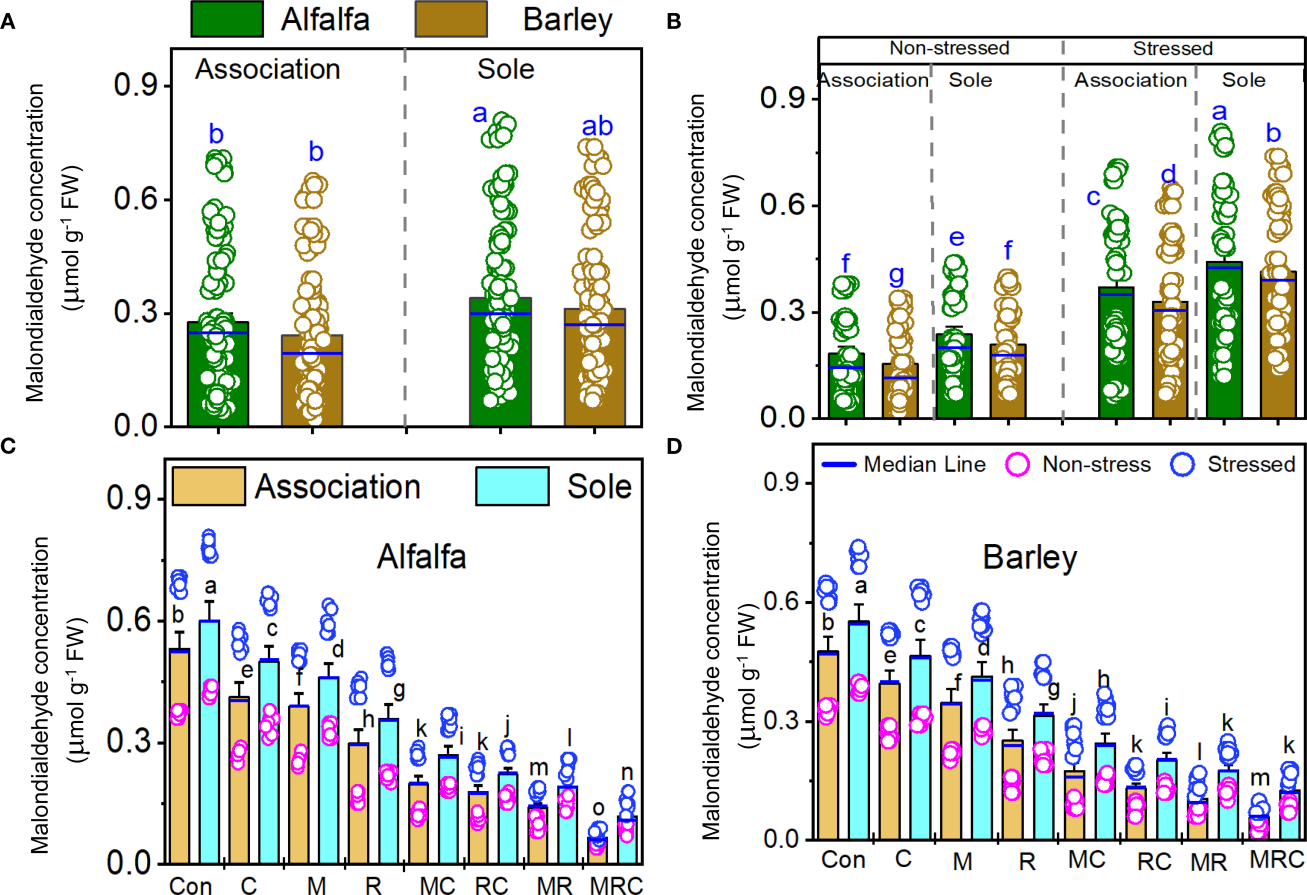
Malondialdehyde concentration of **(A)** alfalfa and barley grown in association or a sole crop, **(B)** stressed and non-stressed of associated and sole cultivation, **(C)** for alfalfa, and **(D)** barley under the different biological treatments. Means (n=8) ± standard error are presented. Means followed by the same letter are not significantly different at *p* < 0.05. Con, control; M, arbuscular mycorrhizal fungi consortium; R, bacterial consortium, C; compost, MC, arbuscular mycorrhizal fungi consortium and compost; RC, bacterial consortium and compost; MR, arbuscular mycorrhizal fungi consortium and bacterial consortium; MRC, arbuscular mycorrhizal fungi consortium, bacterial consortium, and compost.

**Figure 2:**
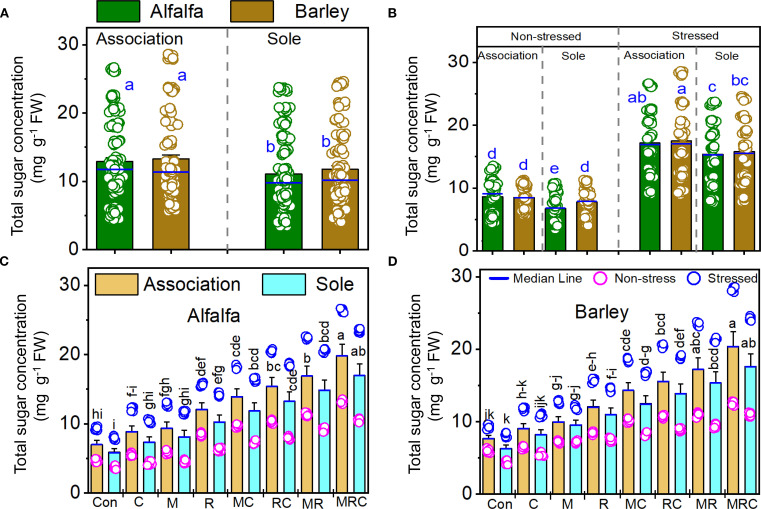
Total sugar contents of **(A)** alfalfa and barley grown in association or a sole crop, **(B)** stressed and non-stressed of associated and sole cultivation, **(C)** for alfalfa, and **(D)** barley under the different biological treatments. Means (n=8) ± standard error are presented. Means followed by the same letter are not significantly different at *p* < 0.05. Con, control; M, arbuscular mycorrhizal fungi consortium; R, bacterial consortium; C, compost; MC, arbuscular mycorrhizal fungi consortium and compost; RC, bacterial consortium and compost; MR, arbuscular mycorrhizal fungi consortium and bacterial consortium; MRC, arbuscular mycorrhizal fungi consortium, bacterial consortium, and compost.

**Figure 3:**
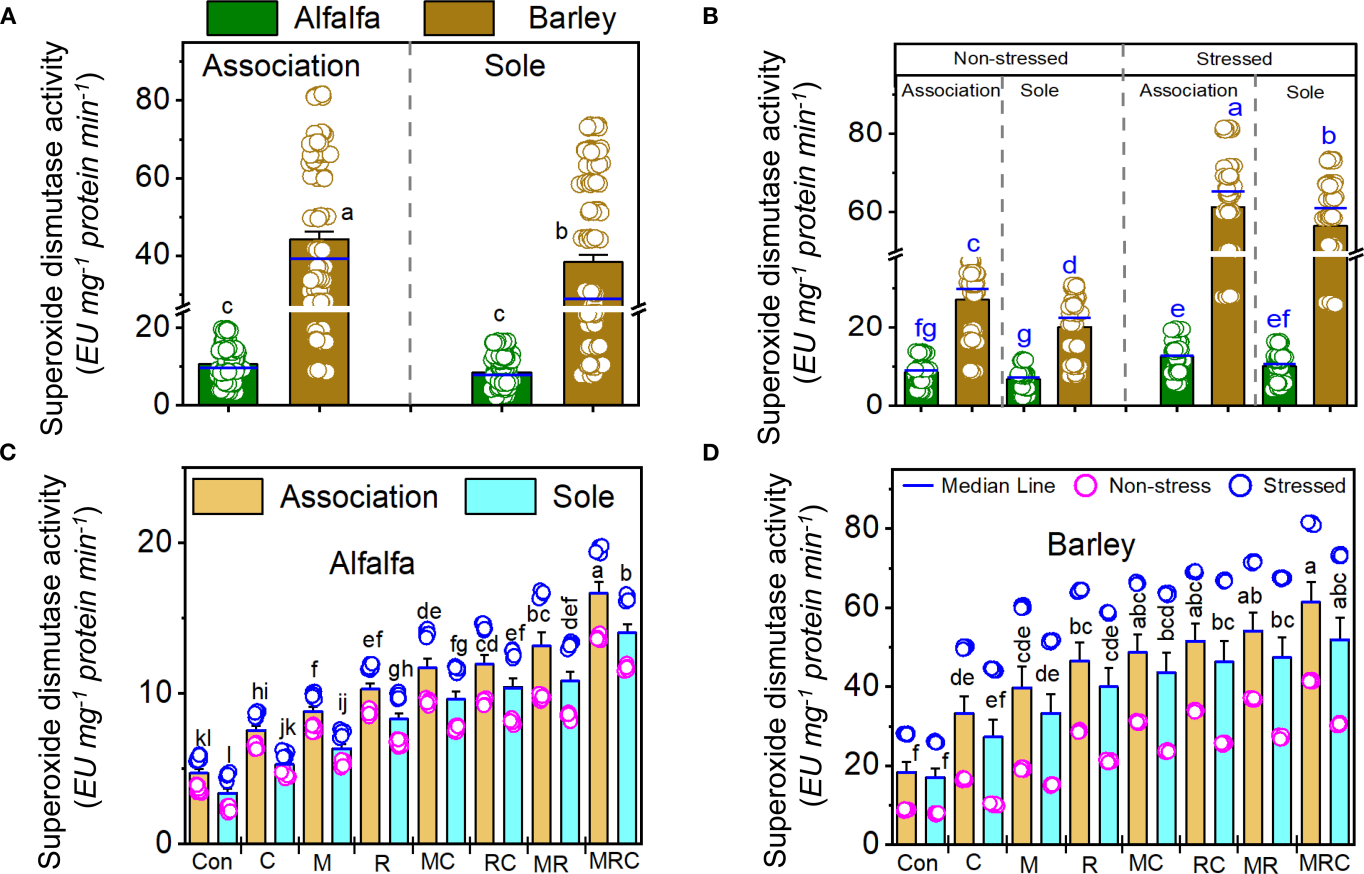
Superoxide dismutase activity of **(A)** alfalfa and barley grown in association or a sole crop, **(B)** stressed and non-stressed, of associated and sole cultivation, **(C)** for alfalfa, and **(D)** barley under the different biological treatments. Means (n=8) ± standard error are presented. Means followed by the same letter are not significantly different at *p* < 0.05. Con, control; M, arbuscular mycorrhizal fungi consortium; R, bacterial consortium; C, compost; MC, arbuscular mycorrhizal fungi consortium and compost; RC, bacterial consortium and compost; MR, arbuscular mycorrhizal fungi consortium and bacterial consortium; MRC, arbuscular mycorrhizal fungi consortium, bacterial consortium, and compost.

The MDA and sugar contents, as well as SOD activity, were significantly (*p* < 0.001) affected by the Es and Bt factors (**Table 4**). The Ca effect was significant on the MDA and sugar contents, but not on SOD activity (**Table 4**). The Es × Bt interaction was significant for both MDA and sugar content. In contrast, Ca × Es was only significant for MDA contentraction was significant for both MDA and sugar content, whereas Ca × Es was only significant for MDA content. The triple interaction Ca × Es ×Bt was significant for the sugar content. The monocropping of alfalfa and barley showed higher MDA levels than the associated crops (**Figure 1A**). Stressed alfalfa in the sole crop treatments exhibited the highest MDA levels compared to other treatment combinations of alfalfa and barley showed higher MDA levels than the associated crops (**Figure 1A**). Stressed alfalfa in the sole crop treatments exhibited the highest MDA levels compared to other treatment combinations (**Figure 1B**). Sole crop of alfalfa and barley under the control treatment showed the highest MDA (**Figures 1C, D**). **Figure 2** displays the sugar content in alfalfa and barley, either grown together or separately, under stressed and non-stressed conditions across various biological combinations. Associated alfalfa and barley showed higher sugar contents compared to plants grown alone (**Figure 2A**). Additionally, the associated alfalfa and barley stressed plants exhibited higher sugar levels than the non-stressed ones (**Figure 2B**). The MRC treatment of alfalfa and barley resulted in higher sugar levels than the other organic and microbiological treatments (**Figures 2C, D**). The expression of SOD activity was higher for barley than for alfalfa. Barley planted in association exhibited significantly higher activity than the sole crop of barley and alfalfa (**Figure 3A**). We found that stressed plants of barley in association recorded the highest activity of SOD (**Figure 3B**). The MRC treatment of alfalfa and barley resulted in higher SOD activity than biologically treated plants (**Figures 3C, D**).

**Table 4:** Three-way analysis of variance (ANOVA) testing the effects of crop association (Ca), environmental stress (Es), biological treatments (Bt), and their interactions on malondialdehyde, sugar content, and superoxide dismutase activity.

Source of variation	DF	Malondialdehyde content (μmol g^-1^ FW)			Sugar content (mg g^-1^ FW)		
		F value	*p* value	Sig.	F value	*p* value	Sig.
Crop association (Ca)	1	904.7	<0.001	(***)	1316.1	<0.001	(***)
Environmental Stress (Es)	1	7658.4	<0.001	(***)	33843.1	<0.001	(***)
Biological treatment (Bt)	7	2533.0	<0.001	(***)	4020.3	<0.001	(***)
Ca × Es	1	30.0	<0.001	(***)	3.1	0.08	ns
Ca × Bt	7	1.4	0.20	ns	22.1	<0.001	(***)
Es × Bt	7	253.5	<0.001	(***)	648.5	<0.001	(***)
Ca × Es × Bt	7	1.0	0.42	ns	4.2	<0.001	(***)

Source of variation	DF	Superoxide dismutase activity (EU mg^-1^ protein min^-^1)					
		F value	*p* value	Sig.			
Crop association (Ca)	1	2.4	0.12	ns			
Environmental Stress (Es)	1	74.9	<0.001	(***)			
Biological treatment (Bt)	7	4.0	<0.001	(***)			
Ca × Es	1	0.02	0.89	ns			
Ca × Bt	7	0.02	1.0	ns			
Es × Bt	7	0.05	1.0	ns			
Ca × Es × Bt	7	0.00	1.0	ns			

DF: degree of freedom. ns, not significant; *Significant at *p* < 0.05; **Significant at *p* < 0.01; ***Significant at *p* < 0.001.

### 3.4 Correspondence analysis (CCA), Pearson correlation, and dendrogram

**Figure 4:**
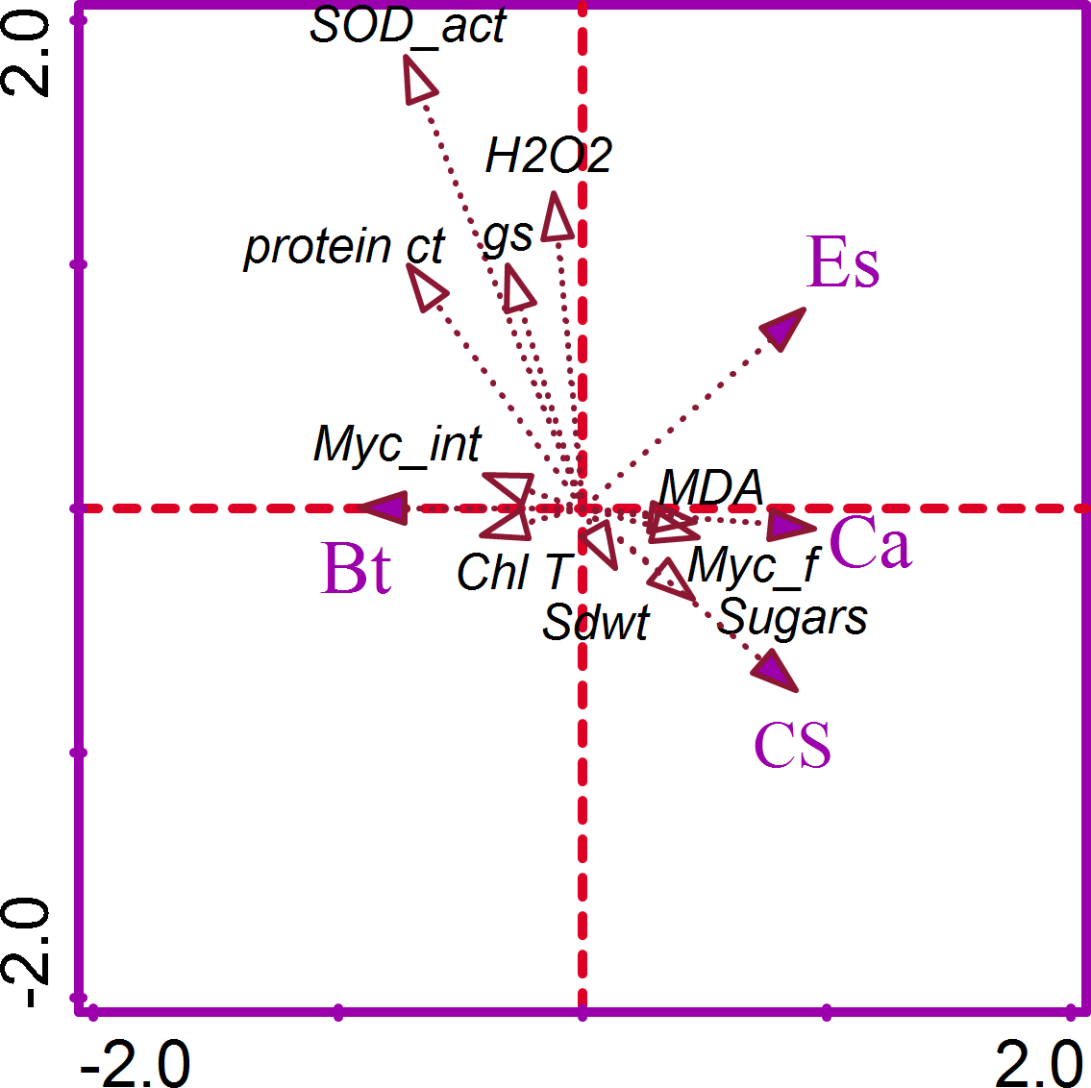
Correspondence analyses of the crop association (Ca), environmental stress (Es), and biological treatments (Bt) on the functional variable measured. Myc_f, mycorrhizal frequency; Myc_int, mycorrhizal intensity; Shoot dwt, total dry weight; gs, stomatal conductance; Chl T, total chlorophyll; H2O2, hydrogen peroxide; MDA, malondialdehyde; SOD, superoxide dismutase; CAT, catalase.

The CCA of the factorial (Ca, Es, Bt) variables and functional traits (gs, Chl T content, mycorrhizal colonization frequency and intensity, shoot growth, H_2_O_2_, MDA, protein and sugar contents, and CAT and SOD activities) was implemented for that dataset (**Figure 4**). The CCA1 and CCA2 axes accounted for 74.2% in explaining the variability in the dataset. The respective contribution of each functional trait to explaining variability in the dataset, along with the *p*-value and adjusted *p*-values, is reported (**Supplementary Table S3**). The Chl T explained 54.4% while the protein content explained 15.0% of the variability in the dataset.

**Figure 5:**
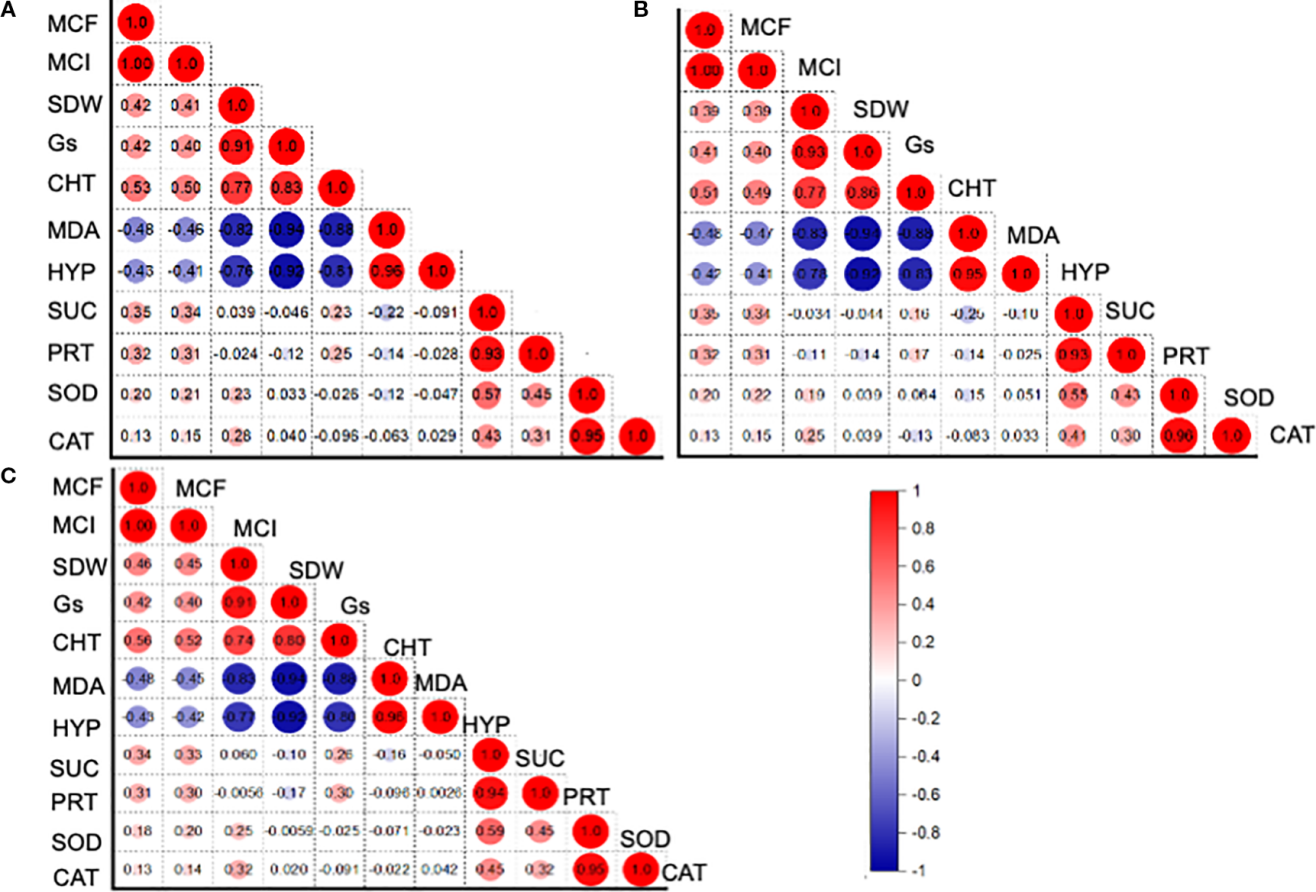
Pearson correlation analysis, describing the relationship between phenotypic, physiological, and biochemical traits for **(A)** entire datasets, **(B)** crop association, and **(C)** for the sole cultivation of alfalfa and barley. MCF: Mycorrhizal colonization frequency (%); MCI: Mycorrhizal colonization intensity (%); SDW: Shoot dry weight (g plant^-1^); Gs: gs (mmol m^-2^ s^-1^); CHC: Ghlorophyll content (g plant^-1^); MAD: Malondialdehyde content (mmol g^-1^ FW); HYP: Hydrogen peroxide (nmol g^-1^ FW); SUC: Sugar content (mg g^-1^ FW); PRT: Protein content (mg g^-1^ FW); SDA: Superoxide dismutase activity (EU mg^-1^ protein min^-1^); CAT: Catalase activity (EU mg^-1^ protein min^-1^).

**Figure 6:**
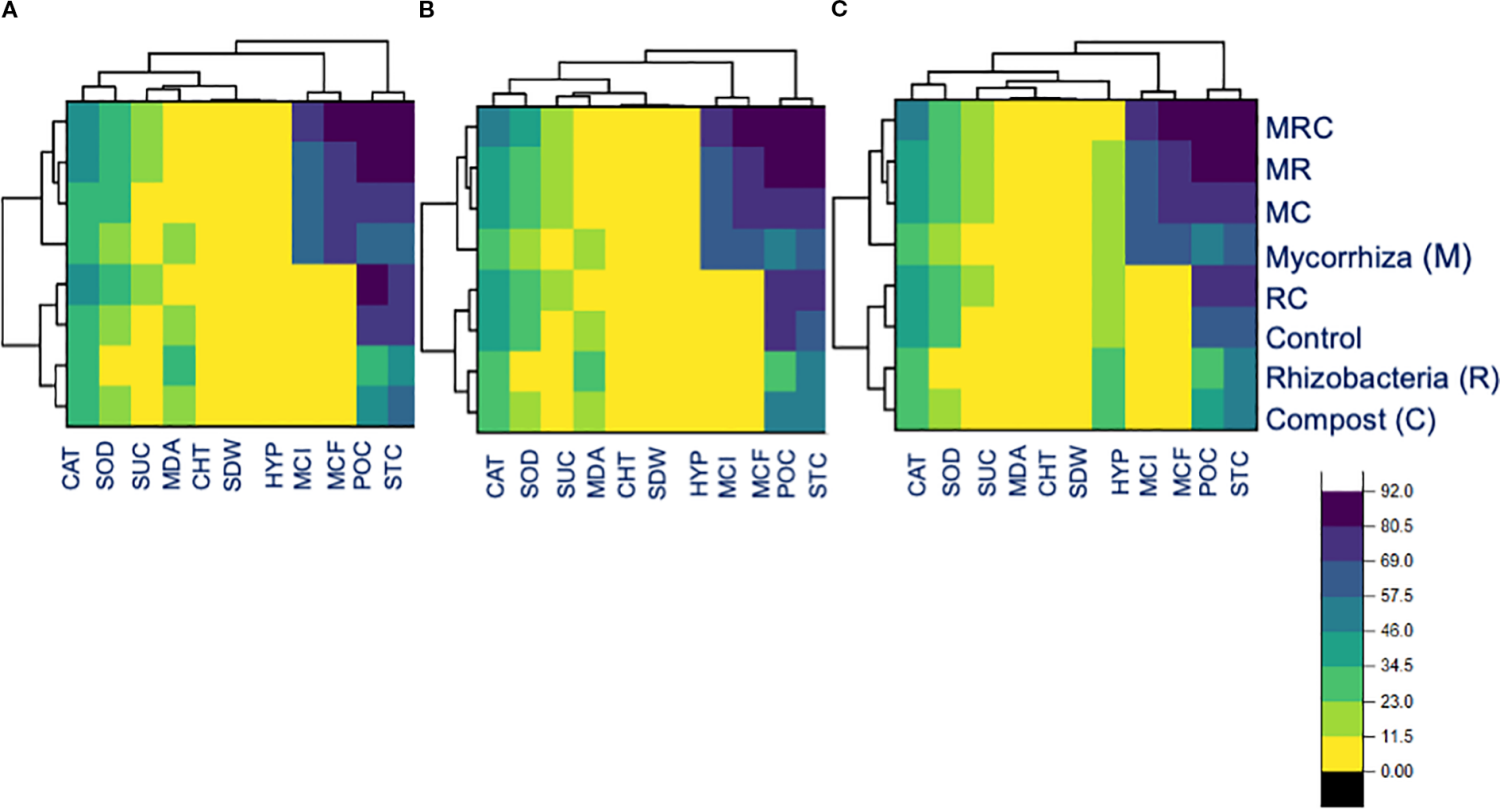
Head map dendrograms based on Euclidean distance and group average values for **(A)** the entire dataset, **(B)** crop association, and **(C)** for the sole cultivation of alfalfa and barley.

The Pearson correlation among the functional variables was computed for the entire dataset, as well as for the associated crop and sole crop (**Figure 5**). For the entire dataset, the relationship (r^2^ = 0.93) between H_2_O_2_ content and protein content was found to be positively significant (**Figure 5A**). Similarly, the there was a positive correlation (r^2^ = 0.95) between the CAT activity and protein content (**Figure 5A**). However, the relationship between MDA and shoot dry weight was negatively correlated (r² = -0.94). A similar trend of creation was observed for the alfalfa and barley association, and this was also evident when grown alone (**Figures 5B, C**). The head map dendrograms, based on Euclidean distance and group average values, were plotted (**Figure 6**). Four clustering groups were identified for the tested functional variables. Cluster 1 was the CAT and SOD activity, while cluster 2 was sugar content, MDA, chl T content, shoot dry weight, and H_2_O_2_ content (**Figures 6A–C**). Cluster 3 included mycorrhizal colonization frequency and intensity, while cluster 4 covered protein content and stomatal conductance, mycorrhizal colonization frequency and intensity, while cluster 4 covered protein content and stomatal conductance. (**Figures 6A–C**). There were slight differences in the phylogenetic trees from the whole dataset, alfalfa and barley association, and sole planting of the two crops (**Figures 6A–C**).

## 4 Discussion

Resilient management of agrosystems in arid and semi-arid zones subjected to drought and salinity can be achieved through a biological approach, such as implementing a legume-cereal association system and low-cost microbial/ecological interventions. We aimed to investigate the potential of crop association practices in conjunction with biological combinations to alleviate drought and salt stress by examining mycorrhization rates, physiological responses, the expression of key stress markers, and the activation of the antioxidant system in plants. We explored the below-ground cascading effects between the associated crops and inoculated microbes. Generally, we found that the association of alfalfa and barley attenuated the negative impacts of drought and salt stress while promoting PGPM effects in mitigating these two most damaging environmental stresses.

### 4.1 Drought and salt stress mitigation benefits of associated cultivation practice

Our findings indicated that the frequency and intensity of mycorrhization in barley and alfalfa were significantly reduced under abiotic stress conditions. However, combining the two cultures increased AMF colonization in both crops. Our results are similar to those obtained by Ingraffia et al. (2019), who observed that intercropping wheat and faba bean improves mycorrhizal colonization more than monocropping. In the crop association system, several factors contribute to regulating AMF colonization, including nutrient transfer and an increase in root density and root exudates, which govern signaling between AMF and host plants, thereby stimulating the activation of AMF symbiosis (Ingraffia et al., 2019). The biomass of both plants significantly decreased under simultaneous drought and salt stress. Under such conditions, plants experience cellular water loss, damage to the plasma membrane, and reduced turgor pressure, which limits cell division and elongation, and slows overall development (Ors et al., 2021). In the mixed cropping system employed, the biomass of barley and alfalfa increased significantly. This system optimizes root and rhizosphere processes through specific interactions that enable resources to be more efficiently mobilized, acquired, and utilized, thereby enhancing biomass production (Li et al., 2024).

The physiological parameters of stressed plants were significantly lower than those of non-stressed plants. This may be due to decreased water availability for the plants, which leads to the closure of leaf stomata, limits transpiration, increases leaf temperature, and reduces the CO_2_ supply to the intercellular space (Murtaza et al., 2024). It also results in the deterioration of rubisco enzymes, the primary key enzymes in plant photosynthesis (Kaur et al., 2021). However, under stressed conditions, stomatal conductance and total chlorophyll content were improved in alfalfa and barley plants grown in a mixed configuration. Crop associations enhance soil moisture and nutrient absorption, resulting in wider stomatal openings (Hussain et al., 2023). This facilitates better light interception by plants and increases carbon assimilation. Consequently, it improves leaf chlorophyll content and strengthens the accumulation of photosynthetic products in the leaves (Hussain et al., 2023).

Under stress, MDA and H_2_O_2_ levels increased significantly in barley and alfalfa leaves. This rise is linked to the harmful impacts of drought and salinity, which compromise cytoplasmic desiccation, disrupt plasma membrane integrity, and alter cellular oxygen metabolism. Consequently, there’s an excess accumulation of ROS, including H_2_O_2_, alongside lipid peroxidation (Ors et al., 2021; Murtaza et al., 2024). However, our study showed that associating alfalfa and barley reduced the concentrations of this oxidative stress marker in both plants. This decrease is attributed to the ability of crop association to improve native soil quality and enhance water and nutrient uptake, leading to overall plant health and resilience (Layek et al., 2018). Furthermore, mixed cropping can alleviate oxidative damage by activating the antioxidant defense system under stress conditions (Melo-Sabogal and Contreras-Medina, 2024).

To maintain cellular hydration, through osmotic adjustment, barley and alfalfa plants accumulate solutes (sugars and proteins) that work as osmolytes and play critical roles in protecting cellular structure integrity. Furthermore, mixed cropping practice significantly improved sugar and protein content in both plants compared to a sole crop. This increase could be attributed to a more significant input of carbon and N during the crop association, which is directly involved in synthesizing these metabolites (Hussain et al., 2023; Li et al., 2024). Additionally, as an adaptive response, plants produce antioxidative enzymes such as SOD and CAT in response to stress to detoxify the effects of ROS. The combined alfalfa and barley significantly increased the activities of these enzymes compared with their respective sole crops. Like our results, Zheng et al. (2022) reported that faba bean-wheat intercropping significantly increased the enzyme activities and expression of the CAT and SOD genes.

### 4.2 Biological combinations to alleviate drought stress

Biological treatments also played a crucial role in mitigating the simultaneous effects of drought and salt stress. Bacterial inoculum and compost significantly enhanced root colonization under stress conditions, especially when plants are intercropped. PGPRs can serve as mycorrhization helper bacteria, improving the root’s receptivity to mycorrhizal colonization, stimulating pre-symbiotic fungal growth, and enhancing root-fungus recognition processes (Awasthi et al., 2024). Compost, containing humic substances, can also stimulate spore germination under stress conditions (Chiasson et al., 2024).

Similarly, microbial and organic treatments enhanced the biomass of both plants under stressed conditions. Phosphorus (P) solubilization, N_2_ fixation, IAA and siderophore production, and ACC deaminase synthesis are standard traits of all the PGPRs utilized and have been recognized as primary contributors to plant biomass promotion. The AMF consortium can also solubilize phosphorus, improve soil structure, and create extensive hyphal networks connected to plant roots, supplying them with water and nutrients while supporting plant growth (Slimani et al., 2024). Additionally, compost enriches the soil with essential nutrients such as N, P, K, calcium, bioactive substances, and humic organic matter, which boosts plant biomass (Rostaei et al., 2024).

Under stressed conditions, the PGPM and compost improved physiological parameters. This increase could result from improved water and nutrient status, primarily N and iron (Fe), due to various combinations of biological treatments. Nitrogen enhances photosynthetic activities and chlorophyll fluorescence in plants due to its direct role in the components of chlorophyll and enzymes (Qi et al., 2021). On the other hand, Fe is an essential component of ribulose 1,5-bisphosphate carboxylase/oxygenase, a crucial enzyme involved in plant photosynthesis (Wang et al., 2017). In line with our results, several studies have demonstrated that using PGPM or compost enhances physiological responses under stress (Hellal et al., 2024; Khan et al., 2024; Singh et al., 2023).

By measuring stress markers, we demonstrated that biological treatments reduced the oxidative stress produced in stressful environments. The decrease in MDA and H_2_O_2_ levels likely occurs because the plants treated with PGPM and compost exhibit defense mechanisms that protect their organelles from oxidative damage by increasing the production of ROS-scavenging antioxidant compounds and enhancing enzymatic antioxidant activities (Al-Turki et al., 2023). Singh et al. (2023) similarly found that microbial inoculation reduced MDA content and membrane permeability under stressed conditions. Talaat and Abdel-Salam (2024) also revealed that effective microorganism, compost, and their interactions decreased the superoxide and hydrogen peroxide content and lipid peroxidation in leaves of wheat plants grown under drought stress.

Sugar and protein contents were increased significantly in the stressed barley and alfalfa plants. The valuable role of PGPM and compost can be attributed to the biological treatment’s ability to improve water status and nutrient concentrations in plants, thereby regulating the levels of total soluble sugars and protein content (Singh et al., 2023). Similarly, our results demonstrated the effectiveness of biological treatments in protecting stressed barley and plants from the detrimental impacts of drought and salt stress. They indicated that they may play a significant role in regulating the production of antioxidants. This improvement can be attributed to the favorable growth conditions provided by the biological treatments, including the availability of N and P, and an increased active Fe content, as CAT and SOD are heme-containing enzymes (Mosalman et al., 2024). Organic amendments and PGPM increased the expression of CAT and SOD genes in plants (Anbuganesan et al., 2024).

### 4.3 Belowground interspecific cascading effects

The effect of PGPM on mitigating water and salt stress was enhanced under the alfalfa/barley association. This indicates that the mixed crop employed specific belowground mechanisms at the rhizosphere level to stimulate the effects of inoculated consortia of microorganisms. Generally, cereal crops receive a significantly higher proportion of N from the soil due to their faster root development. In comparison, legumes aim to compensate for their lower proportion of N in the soil by fixing atmospheric N_2_ through symbiotic relationships with rhizobial species (Duchene et al., 2017). For this reason, legumes initiate chemotaxis to attract specialized rhizobial species, releasing a variety of exudates, including organic acids, enzymes (especially phosphatases and phytases), amino acids, and phytochelatins (also known as phyto-siderophores) (Souid et al., 2023). The production of these exudates leads to the acidification of the rhizosphere and the transformation of unavailable resources into available ones, thus increasing the mobility of mineral elements by making them more soluble and promoting their desorption and transport to plants (Souid et al., 2023). Legume plants induce N transfer to their cereal plant partner. This richness in mineral elements encourages the growth of cereal plants, the proliferation of their root systems, and the development of high above-ground biomass, thereby sequestering more carbon in the soil (Li et al., 2024). This underground investment enhances the soil’s physicochemical quality, enriches soil microbes, and stimulates their activities (Duchene et al., 2017). This leads to the promotion of PGP activities in the rhizosphere, resulting in increased solubilization and absorption of nutrients, as well as the production of phytohormones, from which both plants forming the mixed cropping system benefit (Li et al., 2024). The result is improved plant health and resistance to stress.

## 5 Conclusion

In this investigation, we found that utilizing an alfalfa-barley associating system enhanced the role of PGPM in alleviating the effects of simultaneous drought and salt stress compared to a sole crop. Furthermore, PGPM and compost combinations improved crop resilience to drought and salinity by boosting osmolyte accumulation and stimulating enzymatic antioxidant defense mechanisms. A cascading effect occurs between plant roots and soil microbes during the establishment of symbiotic relationships. This interaction facilitates the release of root exudates, nutrient transfer, and enrichment of soil microorganisms, enhancing the microbes’ PGP activities. Thus, leveraging these beneficial interactions through compatible crop association systems and effective microbial and bioorganic biostimulants can support sustainable agricultural practices in the face of climate change. This strategy provides an ecological and cost-effective approach to managing abiotic stress, thereby enhancing diversity and food production. Complex interactions across multiple interfaces between the roots, rhizosphere, and microbiome lead to cascading effects that significantly enhance the environmental stress-mitigating benefits of PGPM.

## Data Availability

The datasets presented in this study can be found in online repositories. The names of the repository/repositories and accession number(s) can be found in the article/**Supplementary Material**.
