# Supplementary material for: Alfalfa and barley association promote the ability of plant growth-promoting microbes to mitigate drought and salt stresses
Source: Front Plant Sci. 2025 Oct 13;16:1646620. doi: 10.3389/fpls.2025.1646620 (PMC12559892; doi:10.3389/fpls.2025.1646620)
Supplement: Supplementary file 1 [file Table1.docx]

Supplementary Tables

| Table S1: Three-way analysis of variance (ANOVA) testing the effects of crop association (Ca), environmental stress (Es), biological treatments (Bt), and their interactions on the arbuscular mycorrhizal fungi (AMF) colonization frequency and intensity, stomatal conductance (gs), and total chlorophyll (Chl T) content. | | | | | | | |
| --- | --- | --- | --- | --- | --- | --- | --- |
|  | gs (mmol m^-2^ s^-1^) | | | Chl T (mg g^-1^ FW) | | | |
|  | DDL | F value | *p* value | Sig. | F value | *p* value | Sig. |
| Crop association (Ca) | 1 | 359.06 | <0.001 | (***) | 610.8 | <0.001 | (***) |
| Environmental Stress (Es) | 1 | 6191.76 | <0.001 | (***) | 2348.4 | <0.001 | (***) |
| Biological treatment (Bt) | 7 | 663.74 | <0.001 | (***) | 1259.1 | <0.001 | (***) |
| Ca × Es | 1 | 1.14 | 0.29 | ns | 137.4 | <0.001 | (***) |
| Ca × Bt | 7 | 0.27 | 0.97 | ns | 5.5 | <0.001 | (***) |
| Es × Bt | 7 | 23.60 | <0.001 | (***) | 24.7 | <0.001 | (***) |
|  |  | AMF colonization frequency  (%) | | | AMF colonization intensity  (%) | | |
|  |  | F value | *p* value | Sig. | F value | *p* value | Sig. |
| Crop association (Ca) | 1 | 439.1 | <0.001 | (***) | 222.5 | <0.001 | (***) |
| Environmental Stress (Es) | 1 | 277.4 | <0.001 | (***) | 207.3 | <0.001 | (***) |
| Biological treatment (Bt) | 7 | 16025.9 | <0.001 | (***) | 14340.2 | <0.001 | (***) |
| Ca × Es | 1 | 0.93 | 0.33 | ns | 0.85 | 0.36 | ns |
| Ca × Bt | 7 | 64.6 | <0.001 | (**) | 31.9 | <0.001 | (***) |
| Es × Bt | 7 | 40.1 | <0.001 | (**) | 30.4 | <0.001 | (***) |
| Ca × Es × Bt | 7 | 0.43 | 0.88 | ns | 0.32 | 0.95 | ns |

ns: not significant. *: Significant at *p* < 0.05. **: Significant at *p* <0.01. ***: Significant at *p* <0.001

|  | Alfalfa in association | | Sole alfalfa | | Barley in association | | Sole barley | |
| --- | --- | --- | --- | --- | --- | --- | --- | --- |
|  | Non stressed | Stressed | Non stressed | Stressed | Non stressed | Stressed | Non stressed | Stressed |
|  | Total chlorophyll content (mg g^-1^ FW) | | | | | | | |
| Con | 0.83 ± 0.013 | 0.37 ± 0.010 | 0.67 ± 0.012 | 0.24 ± 0.008 | 0.70 ± 0.011 | 0.33 ± 0.008 | 0.49 ± 0.011 | 0.21 ± 0.006 |
| C | 1.17 ± 0.008 | 0.57 ± 0.008 | 0.81 ± 0.007 | 0.46 ± 0.008 | 0.98 ± 0.015 | 0.56 ± 0.001 | 0.75 ± 0.008 | 0.44 ± 0.008 |
| M | 1.50 ± 0.009 | 0.66 ± 0.008 | 0.99 ± 0.011 | 0.53 ± 0.008 | 1.28 ± 0.015 | 0.79 ± 0.008 | 0.93 ± 0.013 | 0.61 ± 0.009 |
| R | 1.99 ± 0.015 | 1.15 ± 0.013 | 1.40 ± 0.012 | 1.00 ± 0.013 | 1.68 ± 0.012 | 0.95 ± 0.008 | 1.24 ± 0.019 | 0.79 ± 0.011 |
| MC | 2.07± 0.012 | 1.49 ± 0.012 | 1.55 ± 0.009 | 1.36 ± 0.008 | 1.78 ± 0.006 | 1.15 ± 0.011 | 1.36 ± 0.009 | 0.99 ± 0.009 |
| MR | 2.77 ± 0.014 | 1.77 ± 0.007 | 1.99 ± 0.011 | 1.56 ± 0.009 | 2.47 ± 0.018 | 1.34 ± 0.011 | 1.98 ± 0.015 | 1.20 ± 0.011 |
| RC | 2.36 ± 0.008 | 1.61 ± 0.008 | 1.84 ± 0.008 | 1.46 ± 0.008 | 2.00 ± 0.011 | 1.27 ± 0.009 | 1.56 ± 0.012 | 1.09 ± 0.008 |
| MRC | 2.93 ± 0.010 | 2.13 ± 0.012 | 2.46 ± 0.009 | 1.96 ± 0.008 | 2.74 ± 0.008 | 1.69 ± 0.012 | 2.31 ± 0.009 | 1.49 ± 0.013 |
|  | Stomatal conductance (mmol m^-2^ s^-1^) | | | | | | | |
| Con | 71.5 ± 0.41 | 34.6 ± 0.32 | 65.7 ± 0.37 | 27.0 ± 0.35 | 77.3 ± 0.35 | 43.1 ± 0.36 | 72.6 ± 0.38 | 37.3 ± 0.32 |
| C | 74.7 ± 0.26 | 44.6 ± 0.38 | 68.9 ± 0.29 | 36.3 ± 0.35 | 83.5 ± 0.27 | 48.1 ± 0.00 | 75.7 ± 0.49 | 42.1 ± 0.37 |
| M | 78.5 ± 0.44 | 48.4 ± 0.44 | 71.4 ± 0.38 | 42.2 ± 0.32 | 86.5 ± 0.45 | 56.0 ± 0.31 | 80.2 ± 0.33 | 46.9 ± 0.46 |
| R | 86.3 ± 0.37 | 55.9 ± 0.39 | 78.4 ± 0.37 | 48.2 ± 0.37 | 94.4 ± 0.36 | 61.8 ± 0.34 | 89.0 ± 0.55 | 52.4 ± 0.26 |
| MC | 90.3 ± 0.33 | 65.2 ± 0.46 | 82.8 ± 0.46 | 56.2 ± 0.36 | 98.4 ± 0.37 | 66.7 ± 0.51 | 93.1 ± 0.40 | 61.3 ±0.32 |
| MR | 96.4 ± 0.39 | 75.7 ± 0.33 | 89.0 ± 0.35 | 67.0 ± 0.33 | 105.4 ± 0.39 | 81.1 ± 0.32 | 98.5 ± 0.40 | 74.1 ± 0.38 |
| RC | 93.5 ± 0.15 | 70.2 ± 0.43 | 85.0 ± 0.35 | 62.2 ± 0.36 | 101.8 ± 0.35 | 74.1 ± 0.38 | 95.2 ± 0.31 | 68.3 ± 0.42 |
| MRC | 99.9 ± 0.30 | 80.5 ± 0.32 | 94.5 ± 0.33 | 72.9 ± 0.34 | 112.5 ± 0.37 | 87.6 ± 0.48 | 105.6 ± 0.40 | 81.6 ± 0.33 |
|  | Mycorrhizal colonization frequency (%) | | | | | | | |
| Con | 0.0 ± 0.00 | 0.0 ± 0.00 | 0.0 ± 0.00 | 0.0 ± 0.00 | 0.0 ± 0.00 | 0.0 ± 0.00 | 0.0 ± 0.00 | 0.0 ± 0.0 |
| C | 0.0 ± 0.00 | 0.0 ± 0.00 | 0.0 ± 0.00 | 0.0 ± 0.00 | 0.0 ± 0.00 | 0.0 ± 0.00 | 0.0 ± 0.0 | 0.0 ± 0.0 |
| M | 77.5 ± 1.27 | 69.2 ± 1.27 | 0.0 ± 1.14 | 56.7 ± 1.31 | 82.5 ± 0.00 | 75.0 ± 1.14 | 70.8 ± 1.27 | 62.5 ± 1.27 |
| R | 0.0 ± 0.00 | 0.0 ± 0.00 | 89.2 ± 0.00 | 0.0 ± 0.00 | 0.0 ± 1.14 | 0.0 ± 0.00 | 0.0 ± 0.00 | 0.0 ± 0.0 |
| MC | 89.2 ± 1.27 | 82.5 ± 1.27 | 68.3 ± 1.31 | 70.8 ± 1.27 | 90.8 ± 1.27 | 83.3 ± 1.31 | 82.5 ± 1.27 | 70.8 ± 1.27 |
| RC | 0.0 ± 0.00 | 0.0 ± 0.00 | 0.0 ± 0.00 | 0.0 ± 0.00 | 0.0 ± 0.00 | 0.0 ± 0.00 | 0.0 ± 0.00 | 0.0 ± 0.0 |
| RC | 0.0 ± 0.00 | 0.0 ± 0.00 | 0.0 ± 0.00 | 0.0 ± 0.00 | 0.0 ± 0.00 | 0.0 ± 0.00 | 0.0 ± 0.00 | 0.0 ± 0.0 |
| MRC | 97.5 ± 1.27 | 88.3 ± 1.14 | 77.5 ± 1.27 | 78.3 ± 1.14 | 98.3 ± 1.27 | 90.8 ± 1.27 | 89.2 ± 1.27 | 84.2 ± 1.27 |
|  | Mycorrhizal colonization intensiy (%) | | | | | | | |
| Con | 0.0 ± 0.00 | 0.0 ± 0.00 | 0.0 ± 0.00 | 0.0 ± 0.00 | 0.0 ± 0.00 | 0.0 ± 0.00 | 0.0 ± 0.00 | 0.0 ± 0.00 |
| C | 0.0 ± 0.00 | 0.0 ± 0.00 | 0.0 ± 0.00 | 0.0 ± 0.00 | 0.0 ± 0.00 | 0.0 ± 0.00 | 0.0 ± 0.00 | 0.0 ± 0.00 |
| M | 66.7 ± 0.46 | 60.8 ± 0.38 | 60.6 ± 0.48 | 55.1 ±0.29 | 73.0 ± 0.50 | 68.0 ± 0.48 | 65.5 ± 0.76 | 59.9 ± 0.48 |
| R | 0.0 ± 0.00 | 0.0 ± 0.00 | 0.0 ± 0.00 | 0.0 ± 0.00 | 0.0 ± 0.00 | 0.0 ± 0.00 | 0.0 ± 0.00 | 0.0 ± 0.00 |
| MC | 71.5 ± 0.38 | 64.9 ± 0.29 | 64.5 ± 0.38 | 58.6 ± 0.48 | 78.9 ± 0.76 | 70.9 ± 0.52 | 71.8 ± 0.85 | 64.5 ± 0.39 |
| RC | 0.0 ± 0.00 | 0.0 ± 0.00 | 0.0 ± 0.00 | 0.0 ± 0.00 | 0.0 ± 0.00 | 0.0 ± 0.00 | 0.0 ± 0.00 | 0.0 ± 0.00 |
| MR | 73.7 ± 0.43 | 64.9 ± 0.29 | 65.1 ± 0.48 | 58.8 ± 0.57 | 78.9 ± 0.43 | 71.5 ± 0.28 | 73.0 ± 0.76 | 67.0 ± 0.48 |
| MRC | 77.5 ± 0.42 | 71.8 ± 0.39 | 71.6 ± 0.57 | 66.1 ± 0.38 | 83.5 ± 0.43 | 77.4 ± 0.31 | 76.4 ± 0.43 | 71.4 ± 0.31 |

Table S2: Effect of crop association, environmental stress, and biological treatments on the total chlorophyll content, stomatal conductance, and mycorrhizal colonization frequency and intensity of alfalfa and barley plants. Means (n=8) ± standard error within the same plant. Con: control, M: arbuscular mycorrhizal fungi consortium, R: bacterial consortium, C: compost, MC: arbuscular mycorrhizal fungi consortium and compost, RC: bacterial consortium and compost, MR: arbuscular mycorrhizal fungi consortium and bacterial consortium, MRC: arbuscular mycorrhizal fungi consortium, bacterial consortium, and compost.

| Table S3: Explained, contribution, pseudo-F, probabilities and adjusted P of the functional variables to canonical axes. | | | | | |
| --- | --- | --- | --- | --- | --- |
| Fonctional trait | Explains % | Contribution % | pseudo-F | P | P(adj) |
| Total chlorophyll content (mg g^-1^ FW) | 54.4 | 62.3 | 607 | 0.0001 | 0.00037 |
| Protein content (mg g^-1^ FW) | 15.0 | 17.2 | 250 | 0.0001 | 0.00027 |
| Shoot dry weight (g plant^-1^) | 9.4 | 10.8 | 225 | 0.0001 | 0.00022 |
| Hydrogen peroxide (nmol g^-1^ FW) | 4.8 | 5.5 | 147 | 0.0001 | 0.00018 |
| Catalase activity (EU mg^-1^ protein min^-1^) | 0.7 | 0.8 | 21.1 | 0.0001 | 0.00016 |
| Superoxide dismutase activity (EU mg^-1^ protein min^-1^) | 1.2 | 1.3 | 39.9 | 0.0001 | 0.00014 |
| Mycorrhizal colonization frequency (%) | 0.5 | 0.5 | 16.1 | 0.0001 | 0.00012 |
| Sugar content (mg g^-1^ FW) | 0.3 | 0.4 | 11.2 | 0.0001 | 0.00014 |
| Malondialdehyde content (μmol g^-1^ FW) | 0.7 | 0.7 | 24.6 | 0.0001 | 0.00011 |
| Stomatal conductance (mmol m^-2^ s^-1^) | 0.3 | 0.3 | 9.7 | 0.0003 | 0.00033 |
| Mycorrhizal colonization intensiy (%) | 0.2 | 0.2 | 8.2 | 0.0003 | 0.0003 |
